# The need for nationally accepted guidelines for undergraduate nuclear medicine teaching in MBChB programmes in South Africa

**DOI:** 10.4102/sajr.v24i1.1874

**Published:** 2020-07-28

**Authors:** Anthonio O. Adefuye, Henry A. Adeola, Stuart More, Zainab Mohamed

**Affiliations:** 1 Division of Health Sciences Education, University of the Free State, Bloemfontein, South Africa; 2 Division of Dermatology, Department of Medicine, Faculty of Health Sciences, Groote Schuur Hospital, University of Cape Town, Cape Town, South Africa; 3 Division of Nuclear Medicine, Department of Radiation Medicine, University of Cape Town, Cape Town, South Africa; 4 Division of Radiation Oncology, University of Cape Town, Cape Town, South Africa

**Keywords:** nuclear medicine, South Africa, medical school curricula, education, undergraduate

## Abstract

According to the South African Health Professions Act No. 56 of 1974, specific skills outcomes of MBChB programmes are that a medical graduate must be able to utilise diagnostic aids, interpret findings and make diagnoses. Imaging techniques are an integral part of the numerous diagnostic and therapeutic aids used in contemporary medical practice; however, in South Africa, no formal directives exist to guide programme directors or nuclear medicine departments regarding an appropriate undergraduate nuclear medicine educational module. As of 2013, six South African schools of medicine are involved in undergraduate nuclear medicine teaching, in which it forms part of clinical modules taught at varying stages in the academic curriculum. Against this backdrop is the inequitable distribution of nuclear medicine resources, training facilities and staffing in the local state health sector. Inadequate undergraduate teaching and provincial differences in nuclear medicine service provision suggest that many clinicians and graduating medical students are unaware of how radionuclide techniques can facilitate patient management. This high level of imaging illiteracy has been associated with lack of patient referral, poor quality and inadequate referral, poor knowledge of radiation doses and poor awareness of radiation risks. Here we highlight the challenges of undergraduate nuclear medicine teaching in South Africa, emphasising the need for the implementation of guidelines for undergraduate nuclear medicine education. Employing nationally accepted guidelines for undergraduate nuclear medicine teaching in South African MBChB programmes will contribute to the effective utilisation of nuclear medicine and molecular imaging as a diagnostic and therapeutic modality by newly qualified medical practitioners.

## Introduction

The practice of medical radiation science (MRS) involves the administration of trace amounts of radionuclides and ionising radiation energy in the diagnosis, treatment and follow-up of medical and surgical pathologic conditions.^1^ The use of electromagnetic radiation led to the creation of the two major medical radiation sub-divisions: diagnostic radiology and clinical radiation therapy.^2^ The discovery of artificial radionuclides led to the development of nuclear medicine (NM).^2^ In its elementary form, the practice of NM involves the injection of radiopharmaceuticals or radiotracers (tracers – compounds labelled with a gamma-ray-emitting or positron-emitting radionuclide) into the body for the purposes of diagnosis. These pharmaceuticals can also be labelled with alpha- or beta-emitting radio-isotopes for targeted radionuclide therapy in a variety of benign and malignant conditions.^1,3^ Nuclear medicine encompasses both diagnostic and therapeutic aspects of disease management and thus differs from other clinical medical imaging modalities. The use of NM in the diagnosis of disease conditions is centred in its ability to detect changes in biological and physiological processes rather than changes in morphology and anatomy.^4,5^

Numerous studies have reported on the high level of imaging illiteracy amongst practising physicians and graduating medical students.^6,7,8^ This has been associated with lack of patient referral,^9^ poor quality and inadequate referral,^10^ poor knowledge of radiation doses for routine imaging procedures,^7^ and poor awareness of radiation risks.^8^

Here, we highlight the challenges of undergraduate nuclear medicine teaching, particularly in South Africa, and emphasise the need for the implementation of guidelines for undergraduate nuclear medicine education in MBChB programmes in South Africa. Employing nationally accepted guidelines for undergraduate nuclear medicine teaching in South Africa will contribute to the effective utilisation of nuclear medicine and molecular imaging as a diagnostic and therapeutic modality by newly qualified medical practitioners.

## Historical perspective of nuclear medicine

The origin of NM can be traced to the discovery of X-rays, radioactivity and radium between 1895 and 1898.^11^ X-rays became the preferred method due to better contrast and speed of obtaining radiographic images. In 1913, George de Hevesy pioneered the tracer principle,^12^ using natural isotopes, which he applied to study biological (plant and animal) systems in 1923.^13^ The first human application of the active radiotracer was carried out by Blumgart and Yens in 1926.^14^ With the invention of the cyclotron by Ernest Lawrence in the 1930s, it became possible to artificially produce radionuclides and obtain images of distribution of radionuclides in the human body.^15^ Benedict Cassen invented the rectilinear scanner in 1951,^16^ while in 1958 Hal Anger invented the predecessor (Anger) camera for the modern gamma cameras.^17^ In the early 1960s, a more effective, revolutionary, metastable nuclear isomer of technetium-99 was produced to replace Iodine-131 (^131^I) for thyroid disorder.^18^

The mathematical algorithms used to reconstruct tomographic images from a set of angular views around the patient led to the invention of PET (positron emission tomography) and SPECT (single photon emission computed tomography) by Phelps et al.^19^ and Kuhl et al.^20^ respectively. From 1980 both PET and SPECT have evolved and become widely available, demonstrating much clinical benefit to justify its continued medical use.^21^ Integrated hybrid imaging combining computed tomography (CT) such as SPECT/CT, PET/CT and PET/magnetic resonance imaging (MRI) have become standard practice in NM.

## Diagnostic and therapeutic applications of radionuclides

Widespread clinical use of NM imaging procedures started in the early 1950s. Nuclear medicine techniques used as diagnostic tools include radioactive iodine scans (or ^99m^Tc pertechnetate scan); octreotide-scans; metaiodo-benzyl-guanidine (MIBG) and parathyroid imaging for endocrinopathies. Positron emission tomography/computed tomography imaging using 2-[18F] fluoro-2-deoxyglucose (^18^F-FDG) has also evolved and it is being utilised in oncological, infective, vascular and neurodegenerative disorders.^22^ Other PET tracers used include [^68^Ga]-DOTA-NOC used for somatostatin receptor imaging in neuroendocrine tumours,^23^ ^68^Ga Prostate Specific Membrane Antigen (PSMA) in prostate cancer,^24^ and ^18^F-Dihydroxyphenylalanine (DOPA) in the imaging of dopaminergic pathways in motor disorders, neuroendocrine tumours (NETs) and congenital hyperinsulinism.^25^ NM imaging procedures are thus an essential part of patient care in most medical specialties.^26^ More recently, with the advent of peptide receptor radionuclide therapy (PRRT), radiopharmaceuticals linked to beta emitters such as Lutetium 177(^177^Lu) in treating neuroendocrine tumours have gained much traction.^3^ However, it should be noted that the diagnostic and therapeutic application of ionising radiation in clinical medicine, should be kept within a safe limit according to the ALARA (as low as reasonably achievable) principle.^27^ It is therefore the responsibility of all healthcare providers to equip themselves with current and appropriate knowledge about radiation safety.

## Global state of undergraduate nuclear medicine education

Over the years, the practice of clinical medicine and surgery has changed dramatically with an increasing reliance on diagnostic tests.^10^ The current era of sophisticated radiological imaging techniques (including nuclear medicine) has put radiation medicine at the forefront of modern medicine with an increasing demand for these services in patient management.^28^ It is therefore essential that medical practitioners and specialists, as well as medical students, be provided with a basic knowledge and understanding of clinical imaging procedures. Medical practitioners should understand the values, procedures, indications, benefits and also the contraindications and financial implications of these technologies in order to optimise their use in improving patient outcomes.^29^

Although radiological imaging has undergone significant changes, these changes are still not fully incorporated and implemented in undergraduate medical school curricula.^30^ Radiological and NM training is considered an adjunct or ancillary subject to clinical modalities and not a ‘core’ subject in medical school curricula.^30^ In a study comprising of 77 European countries, Lass and Scheffler reported that there is a high level of variation in the undergraduate teaching of nuclear medicine across countries, with undergraduate teaching integrated into one of the clinical modules and in some cases presented in clinical physiology.^31,32^ Similarly, using an electronic survey on undergraduate teaching distributed by the European Society of Radiology (ESR) to 38 national delegates of the ESR Education Committee, Kourdioukova et al.^33^ revealed a large number of differences in curriculum content and teaching methods throughout Europe. Furthermore, Moloney et al.^34^ reported a minimal improvement in the knowledge of medical students in requesting radiological investigations over the course of the final medical year in the USA, and this was attributed to a relatively short period of learning in the clinical setting. Moloney and colleagues further suggested that emphasis on education and appropriateness may offer an improvement in the utilisation of radiology services and improve patient care.

### Defining the problem

Despite the obvious clinical importance of imaging and imaging techniques (e.g. Interventional Radiology) in the management of disease conditions in patients, undergraduate medical students receive little or no formal training in clinical imaging (including NM) for which the students must show proficiency by passing a test in the subject.^6^ In most medical colleges, courses in imaging or NM education are presented as electives with no formal assessment.^6,10^ Rogers^6^ stated that medical students often perceive radiology and NM electives as a little vacation from demanding clinical assignments and study, thus leading to a high level of imaging illiteracy amongst graduating medical students.^6^ Commander et al.^35^ showed that 84% (*n* = 217 of 259) of preclinical students and 62% of clinical students (*n* = 110 of 177) at two medical schools in the USA lacked adequate knowledge of interventional radiology. Poorly coordinated and abbreviated periods of learning can have a detrimental effect on patient management as the majority of new medical graduates have limited knowledge of various aspects of imaging and imaging techniques.^36^ Hence, providing medical students with sufficient and precise knowledge regarding different aspects of radiation and NM is a necessity.^36^

### The European Society of Radiology recommendations

According to the White Paper by the European Society of Radiology (ESR) on undergraduate radiological education, a ‘critical core’ curriculum for undergraduate radiological imaging was recommended to be integrated across the existing medical curricula.^37^ The ESR also suggested that medical schools should ensure that such an important undergraduate imaging ‘core’ curriculum must be delivered to students according to outcome-based education (OBE) strategies. They suggested that a ‘core plus’ curriculum option might also be included.^37^ It is therefore essential for the radiology/NM departments in various colleges of medicine to contribute to the education of the next generation of medical doctors, and no longer ‘willingly cede’ teaching of clinical imaging procedures to other clinical disciplines involved in medical education.^38^ The Alliance of Medical Student Educators in Radiology (AMSER) has also recommended core topics within nuclear medicine that need to be covered during the training of undergraduate medical students.^39^

## The South African perspective

Medical education in South Africa commenced in 1912 in Cape Town, South Africa, after the first medical school opened its doors in 1900. Undergraduate medical education in South Africa is offered at eight Schools of Medicine located within campus-based universities.^40^ According to Burch,^41^ all South African Schools of Medicine have undertaken major curriculum reform over the past 10 years. Despite curriculum differences, the exit-level outcomes for healthcare professionals are the same, as prescribed by the various regulatory bodies. The introduction of the outcome-based education (OBE) approach to education in South Africa brought about major changes in the traditional way teachers approach the teaching process.^42^ This educational strategy revolves around learner-centred, outcome-orientated activities. Emphasis is placed on an integrated teaching and training approach, human development and lifelong learning.^43^

Specific skills outcomes of MBChB programmes as described by the Health Professions Act 56 of 1974 (Act No. 56 of 1974) are that a medical graduate must be able to utilise diagnostic aids, interpret findings and make diagnoses.^44^ Imaging and imaging techniques are an integral part of the numerous diagnostic and therapeutic aids used in contemporary medical practice. However, no formal directives exist in South Africa to guide programme directors or nuclear medicine departments with regard to an appropriate undergraduate medical nuclear medicine educational module.^10^ Six schools of medicine in South Africa are currently involved in undergraduate nuclear medicine education,^10,45^ where it is taught as part of clinical modules at varying stages in the academic curriculum.^10^ These differences in medical curricula and the absence of structured undergraduate nuclear medicine education in two schools of medicine implies that newly qualified doctors with limited or no exposure to undergraduate nuclear medicine teaching may lack the necessary knowledge, skills and right attitude towards imaging modalities and how to use them appropriately to solve patients’ problems. This is evident in the poor quality and inadequate referrals sent by newly qualified medical interns and some community-service doctors to nuclear medicine departments.^10^ In a survey done to ascertain the knowledge of interns and registrars working at two academic hospitals (Charlotte Maxeke Johannesburg Academic Hospital and Chris Hani Baragwanath Academic Hospital) on the basic principles and clinical applications of NM, Dhoodhat et al.^46^ reported that only 41% of the cohort had undergraduate nuclear medicine exposure. According to Dhoodhat and colleagues, more than half of the respondents (52%) had less than 5 h of NM training in their entire curriculum and only 9.9% of them deemed their undergraduate NM education exposure to have been sufficient.^46^ Their findings also showed that the mean NM knowledge score of those participants who had undergraduate NM exposure was significantly higher than those who had no exposure. They also commented on the need to improve undergraduate NM training in South Africa.^46^ Similarly, in a study done to investigate doctors’ awareness of diagnostic radiation exposure at Dr George Mukhari Academic Hospital Ga-Rankuwa, Pretoria, Dauda et al.^8^ reported that 80% of the 217 participants had no formal training on radiation exposure. Dauda and colleagues also advocated for improved teaching on radiation medicine at undergraduate level.^8^

At present, there is only one unpublished study on the development of a guideline for teaching and learning nuclear medicine in undergraduate medical education in South Africa that could be useful in curriculum development.^10^ However, the guidelines proposed by the author of this study have never been implemented.

## Conclusion

In conclusion, this article highlights the challenges of undergraduate nuclear medicine education, more particularly in South Africa, and recognises the need for the implementation of formal guidelines for undergraduate medical nuclear medicine education in MBChB programmes in South Africa. The present status quo, i.e. limited undergraduate teaching, suggests that clinicians are not as knowledgeable on how radionuclide techniques can help in the management of patients.^9^ Therefore, patients who could benefit from these procedures are not referred.^9^ We hope that this article will generate a wider and more robust conversation between curriculum planners at the various universities and the nuclear medicine community as a whole on the need for a holistic (countrywide) study on the development of a national guideline for teaching nuclear medicine in undergraduate medical education in South Africa. Implementation of nationally accepted guidelines for undergraduate medical nuclear medicine education in South Africa will contribute to the effective utilisation of nuclear medicine and molecular imaging as a modality by newly qualified medical practitioners (interns and community-service doctors) in the country.

### Recommendations

In order to change the present status quo and enhance the teaching and learning of NM in the undergraduate MBChB programmes in South Africa, the researchers propose the following implementable recommendations:

- **Form a nationwide consortium to investigate or research the educational needs and current challenges of teaching NM in undergraduate medical education in South Africa:** Such a study should obtain perspectives of students, educators, curriculum planners and deans of the various medical schools in the country. Recommendations made can inform the development of nationwide guidelines.
- **Develop nationwide guidelines for teaching and learning NM in undergraduate medical education in South Africa:** Curriculum planners at the various universities and the various NM or radiation medicine departments countrywide can do this. Such guidelines should be internationally benchmarked, locally relevant and must be approved by the regulatory bodies in charge of undergraduate medical training (i.e. Health Professions Council of South Africa and the Department of Higher Education and Training). These guidelines can inform the development of teaching and learning outcomes, as well as the mode of teaching.
- **Development of a Case-Based Learning (CBL) module for NM:** A CBL module can be developed and integrated into the current MBChB curricula. The integration of CBL into the current MBChB curricula provides medical students with the opportunity to learn NM in a practical and interactive manner,^47^ and avert unnecessary lengthening of training duration.
- **Develop a training-platform-sharing model:** Medical colleges with a well-established training platform should be encouraged to share their training-platforms with colleges lacking such training platforms. This can be arranged as directed electives.
